# *Macleaya cordata* Alkaloids Sanguinarine and Chelerythrine Inhibit *Nocardia seriolae* by Disrupting Cell Envelope Integrity and Energy Metabolism: Insights from Transcriptomic Analysis

**DOI:** 10.3390/microorganisms13122790

**Published:** 2025-12-08

**Authors:** Lei Huang, Xue Cai, Kuan Chu, Xuemei Yuan, Xianqi Peng, Jing Chen, Xialian Bu, Chen Niu, Dawei Song, Jiayun Yao

**Affiliations:** 1 Key Laboratory of Healthy Freshwater Aquaculture, Ministry of Agriculture and Rural Affairs, Key Laboratory of Fish Health and Nutrition of Zhejiang Province, Key Laboratory of Fishery Environment and Aquatic Product Quality and Safety of Huzhou City, Zhejiang Institute of Freshwater Fisheries (Zhejiang Freshwater Fishery Environmental Monitoring Station), Huzhou 313000, China; 2 Life Science College, Huzhou University, Huzhou 313000, China

**Keywords:** *Nocardia seriolae*, *Macleaya cordata* alkaloids, antibacterial activity, transcriptome, antibiotic alternatives

## Abstract

*Nocardia seriolae* infection poses a serious threat to largemouth bass (*Micropterus salmoides*) aquaculture, owing to the lack of effective control strategies. This study investigated the antibacterial effects and underlying mechanisms of *Macleaya cordata* alkaloids—sanguinarine (SE) and chelerythrine (CHE)—against *N*. *seriolae* through integrated physiological and transcriptomic approaches. Results showed SE and CHE exhibited strong in vitro antibacterial activity, with minimum inhibitory concentrations (MICs) of 62.5 and 7.8 μg/mL, respectively. In vivo trials revealed that dietary supplementation with either alkaloid significantly enhanced the survival of infected fish, yielding relative percent survival (RPS) values of 34.5% for SE and 40.0% for CHE. Concurrently, both treatments reduced bacterial load and alleviated granulomatous pathology in multiple organs, including the liver, spleen, and kidney. Physiological analyses revealed severe damage to the cell envelope, as evidenced by increased membrane permeability and structural disintegration observed under transmission electron microscopy (TEM). Transcriptomic profiling identified 3708 and 5095 differentially expressed genes (DEGs) in the SE- and CHE-treated groups, respectively, with notable downregulation of key genes involved in peptidoglycan biosynthesis, the citrate cycle, oxidative phosphorylation, and the pentose phosphate pathways. These findings demonstrate that SE and CHE inhibit *N. seriolae* through a multi-target mechanism simultaneously disrupting cell envelope integrity and energy production, laying the groundwork for their development as eco-friendly aquaculture therapeutics.

## 1. Introduction

Nocardiosis caused by *N. seriolae* poses a severe threat to largemouth bass (*M. salmoides*) aquaculture. This disease is characterized by chronic progression, high infectivity, and substantial mortality, leading to significant economic losses [1]. The pathogen invades host fish, inducing tissue necrosis and granuloma formation—nodular lesions containing bacterial aggregates and necrotic debris [2,3]. As an intracellular parasite, *N. seriolae* evades immune clearance by phagocytes and survives within macrophages [4]. It utilizes these cells as vehicles for systemic dissemination, which complicates therapeutic interventions. Current disease management relies predominantly on conventional antibiotics, yet their efficacy is limited by poor intracellular penetration and emerging drug resistance. Furthermore, antibiotic overuse exacerbates problems such as the proliferation of drug-resistant strains, food safety risks, and ecological disruption, underscoring the urgent need for sustainable alternatives.

Plant-derived compounds from traditional Chinese medicine present promising candidates, characterized by natural biodegradability, low residual toxicity, and reduced potential for inducing antimicrobial resistance [5]. Among these, *M. cordata* stands out for its rich content of benzophenanthridine alkaloids, notably SE and CHE, which exhibit broad-spectrum antibacterial, anti-inflammatory, and antitumor activities [6]. Previous studies have confirmed their efficacy against various Gram-positive and Gram-negative bacteria, such as *Staphylococcus aureus* and *Vibrio parahaemolyticus* [7,8]. These alkaloids employ a multi-target antibacterial strategy, including disrupting cell membrane integrity, impairing biofilm formation, and interfering with essential metabolic processes. Evidence shows that SE and CHE inhibit the growth of pathogens like *S. aureus* and *Streptococcus agalactiaeby* compromising the permeability of the cell envelope and inducing reactive oxygen species (ROS) production [9,10]. Furthermore, the combination of CHE and SE also effectively inhibits the adhesion and biofilm formation of fungal pathogens including *Candida albicans* and *Cryptococcus neoformans* [11]. However, existing research has primarily focused on morphological damage, and the global molecular mechanisms—particularly at the transcriptional level—remain largely unexplored in *N. seriolae*.

Advances in transcriptomic and proteomic technologies have enabled deeper insights into host–pathogen interactions and antibacterial mechanisms. For instance, integrated multi-omics studies have revealed immune regulatory mechanisms in aquaculture species such as largemouth bass and snakehead in response to *N. seriolae* infection [12,13,14,15]. Concurrently, transcriptomic approaches have demonstrated that certain antimicrobial drugs inhibit bacterial pathogens by modulating genes associated with biofilm formation and flagellar assembly [16,17]. Nevertheless, the transcriptional responses of *N. seriolae* to benzophenanthridine alkaloids have not yet been systematically investigated. To address this knowledge gap, the present study integrated physiological and transcriptomic approaches to systematically investigate the antibacterial effects of SE and CHE against *N. seriolae*. This integrated approach aims to elucidate the antibacterial mechanisms of *M. cordata* alkaloids and support their development as sustainable therapeutics for aquaculture.

## 2. Materials and Methods

### 2.1. Bacterial Strain and Reagents

*N. seriolae* strain 21811, stored at –80 °C, was revived and cultured in Brain Heart Infusion broth (BHI) at 28 °C with shaking at 180 rpm for 5 days to obtain mid-logarithmic phase cells for subsequent assays. SE and CHE were purchased from Chengdu Mansite Biotechnology Co., Ltd. (Chengdu, China). Resazurin and other biochemical reagents were obtained from commercial sources and were of analytical grade. Stock solutions of SE and CHE were prepared in dimethyl sulfoxide (DMSO) and serially diluted with BHI medium to achieve the desired test concentrations, with the final DMSO concentration not exceeding 1% (*v*/*v*) in all experiments.

### 2.2. In Vitro Antibacterial Activity Assays

#### 2.2.1. Determination of Minimum Inhibitory and Bactericidal Concentrations

The MICs of SE and CHE against *N. seriolae* were determined using the modified resazurin microtiter assay [18]. Briefly, bacterial suspensions (1 × 10^5^ CFU/mL) were exposed to serial two-fold dilutions of SE and CHE in 96-well plates. Wells containing 1% DMSO in BHI broth served as the negative control, while wells with bacterial suspension in BHI broth without drugs served as the positive control. After incubation at 28 °C for 5 days, 50 μL of resazurin solution (0.01%) was added to each well, and color development was assessed after additional 24 h incubation. The MIC was defined as the lowest concentration that prevented color change from blue to pink. For MBC determination, 100 μL aliquots from wells showing no bacterial growth were plated on BHIA and incubated for 5 days. The MBC was defined as the lowest concentration that resulted in no colony formation. All assays were performed in triplicate.

#### 2.2.2. Growth Curve Analysis

*N. seriolae* cultures (1 × 10^7^ CFU/mL) were treated with SE or CHE at final concentrations corresponding to their respective MICs (62.5 and 7.8 μg/mL), 1/2 MICs (31.3 and 3.9 μg/mL), and 1/4 MICs (15.6 and 2.0 μg/mL). Untreated cultures served as controls. All samples were incubated at 28 °C with shaking at 180 rpm, and bacterial growth was monitored by measuring OD_600_ every 2 days for 14 days using a spectrophotometer. Three biological replicates were performed for each condition.

### 2.3. In Vivo Antibacterial Assays

#### 2.3.1. Experimental Animals and Experimental Design

Healthy largemouth bass with an average body weight of 11.2 ± 1.7 g were obtained from a local fish farm. Prior to experimentation, fish were acclimatized for two weeks in 300 L fiberglass tanks with continuous aeration, maintaining water temperature at 25 ± 1 °C, pH at 7.2 ± 0.2, and dissolved oxygen above 5.0 mg/L. During this period, fish were fed twice daily with a commercial basal diet (crude protein ≥ 45%, crude fat ≥ 8%). After acclimation, fish were experimentally infected via intraperitoneal injection with *N. seriolae* suspended in sterile phosphate-buffered saline (PBS) at a concentration of 1 × 10^7^ CFU/fish. The control group received an equivalent volume of sterile PBS. Following challenge, infected fish were randomly distributed into three groups (150 fish per group): (1) infected control group (CK, fed basal diet), (2) SE-treated group (basal diet supplemented with 1 g SE/kg feed), and (3) CHE-treated group (basal diet supplemented with 1 g CHE/kg feed). The supplementation level of 1 g/kg was selected based on a preliminary tolerance trial with healthy largemouth bass. Each group was maintained in separate tanks with five replicates (30 fish per replicate). The feeding trial was conducted for 35 days, with fish fed twice daily at 3% of body weight. Throughout the acclimatization and experimental periods, no adverse effects on behavior, feeding activity, or gross morphology were observed in fish from any group.

#### 2.3.2. Sample Collection and Analysis

The protective efficacy of SE and CHE was evaluated after 35 days of treatment based on multiple indicators including RPS, bacterial load, clinical signs, and histopathological changes. At the endpoint of the experiment, liver tissues were aseptically collected for bacterial load quantification using a TaqMan qPCR method specific for *N. seriolae*, as previously established by Zhou Ke-Xing et al. based on the *secA* gene [19]. Mortality was recorded daily throughout the 35-day experimental period, and RPS was calculated as [1 − (% mortality in treated group/% mortality in control group)] × 100. For pathological assessment, liver, spleen, kidney, and head kidney tissues were examined for gross lesions, with particular attention to characteristic white granulomatous nodules. Tissues were then processed for histological examination through fixation in 10% neutral buffered formalin, paraffin embedding, sectioning, and hematoxylin-eosin staining to evaluate granulomatous inflammation and necrotic changes.

### 2.4. Cell Membrane Permeability and Ultrastructural Analysis

#### 2.4.1. Membrane Permeability Assessment

Bacterial suspensions of *N. seriolae* were adjusted to a density of 1 × 10^7^ CFU/mL and treated with 1/4 MIC of SE or CHE, while an untreated suspension served as the control (CK). This sub-inhibitory concentration was selected to probe physiological disruptions without causing complete bacteriolysis. All groups were incubated at 28 °C for 5 days. Cells were collected by centrifugation at 8000 rpm for 5 min at 4 °C. The β-D-galactosidase activity was determined using the ONPG method [20]: bacterial cells were collected, resuspended in phosphate buffer (0.1 M, pH 7.0) containing 1.5 mM ONPG, and incubated at 37 °C for 30 min. The reaction was terminated with 1 M Na_2_CO_3_, and the absorbance was measured at 420 nm. For extracellular protein quantification, supernatants were collected following the same centrifugation protocol and analyzed using the Bradford method [21]. Absorbance was measured at 595 nm, and protein concentration was calculated based on the standard curve. All assays were performed in triplicate.

#### 2.4.2. Transmission Electron Microscopy

After 3 days of treatment with 1/4 MIC of SE or CHE, bacterial cells were collected by centrifugation at 8000 rpm for 5 min. The pellets were fixed with 2.5% glutaraldehyde in 0.1 M phosphate buffer (pH 7.4) at 4 °C overnight, then post-fixed with 1% osmium tetroxide for 2 h. Samples were dehydrated through a graded ethanol series (30%, 50%, 70%, 80%, 90%, 95%, and 100%), embedded in epoxy resin, and polymerized at 60 °C for 48 h. Ultrathin sections (70 nm) were prepared using an ultramicrotome, stained with uranyl acetate and lead citrate, and observed under a transmission electron microscope (HT7800, Hitachi, Japan) at 80 kV.

### 2.5. Transcriptome Analysis

#### 2.5.1. Sample Preparation, RNA Extraction and Sequencing

*N. seriolae* suspensions (1 × 10^7^ CFU/mL) were treated with 1/4 MIC of SE or CHE in BHI broth to capture transcriptomic responses under a mechanistically informative, growth-inhibitory condition. Control groups received equivalent volumes of sterile BHI medium. Each treatment group included five biological replicates. All groups were incubated at 28 °C for 3 days with shaking at 180 rpm. After incubation, bacterial cells were collected by centrifugation at 8000 rpm for 3 min, flash-frozen in liquid nitrogen, and stored at −80 °C. Total RNA was extracted using TRIzol reagent. RNA concentration and purity were determined using a Nanodrop2000 spectrophotometer, and integrity was confirmed by agarose gel electrophoresis. After ribosomal RNA depletion, sequencing libraries were constructed and sequenced on the NovaSeq X Plus platform (Illumina, San Diego, CA, USA) to generate 150 bp paired-end reads.

#### 2.5.2. Bioinformatic Analysis

Raw sequencing reads were quality-controlled using fastp to obtain clean reads. Clean reads were aligned to the *N. seriolae* RIMD 2210633 reference genome using Bowtie2 [22], and gene expression levels were quantified using RSEM [23]. Principal component analysis (PCA) was performed to visualize global transcriptomic differences among groups. Venn analysis was conducted to identify unique and shared amplicon sequence variants across groups. Differential expression analysis was performed using DESeq2 [24] with thresholds of |log_2_(fold change)| > 1 and an adjusted *p*-value (False Discovery Rate, FDR) < 0.05. The results of differential expression were visualized using volcano plots. KEGG pathway enrichment analysis of differentially expressed genes (DEGs) was performed using clusterProfiler with a significance threshold of adjusted *p*-value < 0.05. Hierarchical clustering analysis of DEGs involved in energy metabolism and cell wall biosynthesis pathways was conducted using the pheatmap package in R.

### 2.6. Quantitative Real-Time PCR Validation

To validate the reliability of the RNA-seq data, eight DEGs involved in cell wall integrity, energy metabolism, transport and virulence, were selected for quantitative real-time PCR (qRT-PCR) analysis. Gene-specific primers were designed using the Primer-BLAST tool (version 2.0) on the NCBI website (Table 1) and synthesized by Zhejiang Shangya Biotechnology Co., Ltd. (Hangzhou, China). Total RNA extracted from bacterial samples was treated with DNase I (TaKaRa) to remove genomic DNA contamination, and then reverse transcribed into cDNA using a PrimeScript RT reagent kit. qRT-PCR was performed in a 10 μL reaction system containing 5 μL of 2× Taq Pro Universal SYBR qPCR Master Mix, 0.4 μL of each forward and reverse primer, 1 μL of cDNA template, and 3.2 μL of ddH_2_O. The amplification protocol consisted of initial denaturation at 95 °C for 10 min, followed by 45 cycles of 95 °C for 10 s, 55 °C for 10 s, and 72 °C for 30 s. The 16S rRNA gene was used as the internal reference, and each sample was analyzed in three technical replicates. Relative expression levels were calculated using the 2^−ΔΔCt^ method.

### 2.7. Statistical Analysis

All experimental data are presented as mean ± standard deviation. Statistical analyses were performed using SPSSPRO 1.1.5, with one-way ANOVA followed by Tukey’s test for multiple comparisons. A *p*-value < 0.05 was considered statistically significant.

## 3. Results

### 3.1. In Vitro Antibacterial Activity

**Figure 1:**
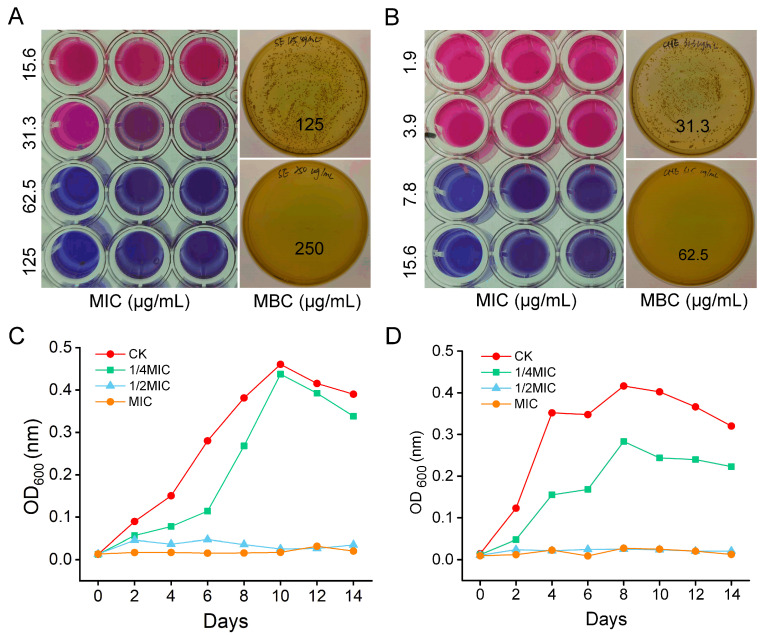
In vitro inhibitory effects of SE and CHE against *N. seriolae.* (**A**) MIC and MBC of SE. (**B**) MIC and MBC of CHE. (**C**) *N. seriolae* growth curves under SE treatment at varying concentrations. (**D**) *N. seriolae* growth curves under CHE treatment at varying concentrations. CK: untreated control group.

The MICs of SE and CHE against *N. seriolae* were determined using the resazurin reduction assay. Bacterial suspension in the 96-well plates exhibited a blue color change (indicating no viable bacteria) at SE concentrations > 62.5 μg/mL and CHE concentrations > 7.8 μg/mL (Figure 1A,B), indicating these concentrations as the respective MICs. MBCs were determined by subculturing from wells showing no growth (≥MIC). No colony formation was observed following incubation for cultures treated with SE > 250.0 μg/mL or CHE > 62.5 μg/mL (Figure 1A,B), defining these concentrations as the respective MBCs. Bacterial growth curves, monitored by measuring OD_600_ over a 14-day period, revealed significantly lower optical densities in all drug-treated groups (MIC, 1/2 MIC, 1/4 MIC) compared to the untreated CK group. Furthermore, a concentration-dependent decrease in OD600 was evident. Notably, cultures treated with 1/2 MIC or MIC of either drug maintained consistently low OD_600_ values (<0.1) throughout the incubation period (Figure 1C,D). These results indicate that both SE and CHE effectively inhibited the growth of *N. seriolae* at concentrations ≥ 1/4 MIC.

### 3.2. In Vivo Antibacterial Effects

**Figure 2:**
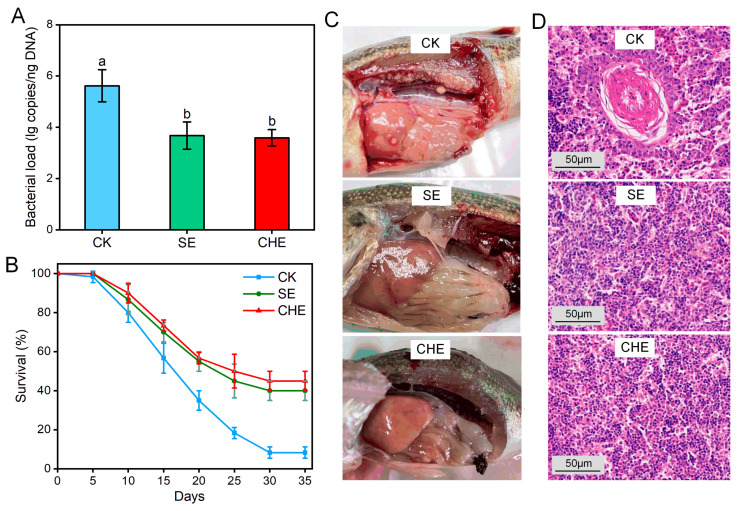
In vivo inhibitory effects of SE and CHE on *N. seriolae*-infected largemouth bass. (**A**) Bacterial load in liver. Data present means ± standard deviations (*n* = 3). Different lowercase letters on the histogram indicate a significant difference (one-way ANOVA followed by Tukey’s post hoc test, *p* < 0.05). (**B**) Survival rate. (**C**) Clinical symptoms. (**D**) Histopathology of head kidney (CK group showing granuloma formation).

Largemouth bass experimentally infected with *N. seriolae* were fed diets supplemented with SE or CHE for 35 days to assess in vivo antibacterial efficacy. Bacterial load analysis revealed that the levels of *N. seriolae* in the liver was significantly lower (*p* < 0.05) in both SE- and CHE-treated groups compared to the untreated CK group after 35 days (Figure 2A). Conversely, survival rates were markedly higher in SE-treated (40.0 ± 5.0%) and CHE-treated (45.0 ± 5.0%) groups compared to the CK group (8.3 ± 2.9%) (Figure 2B), yielding RPS values of 34.5% and 40.0%, respectively. Gross pathology at day 35 revealed characteristic white granulomatous lesions in the liver, spleen, and kidney of CK fish, while no obvious lesions were observed in SE- or CHE-treated fish (Figure 2C). Similarly, histopathology of the head kidney confirmed granulomatous necrosis in the CK group, whereas no significant pathological alterations were detected in treated groups (Figure 2D).

### 3.3. Effects of SE and CHE on Cell Structure of N. seriolae

**Figure 3:**
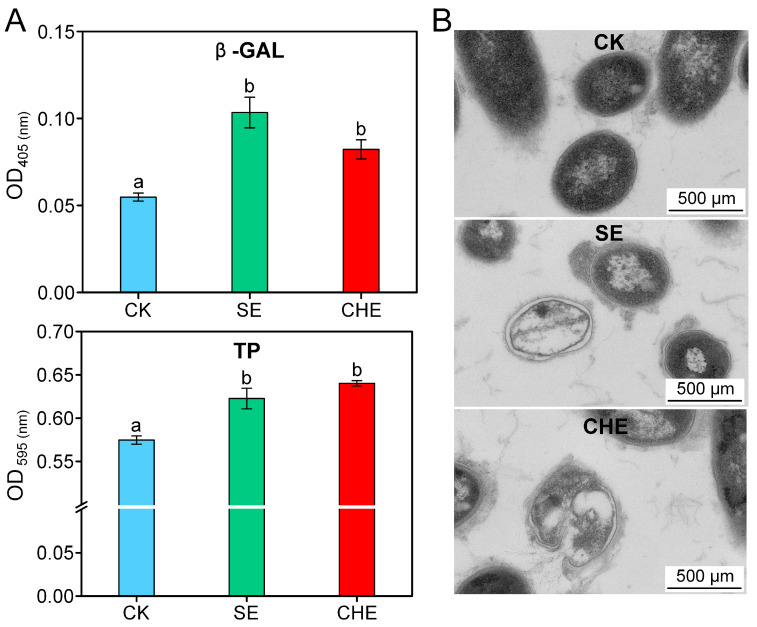
Impact of SE and CHE on *N. seriolae* cell integrity. (**A**) Cell membrane permeability assessed by β-galactosidase (β-GAL) activity and total protein (TP) release. CK: untreated control group. Data present means ± standard deviations (*n* = 3). Different lowercase letters on the histogram indicate a significant difference (one-way ANOVA followed by Tukey’s post hoc test, *p* < 0.05). (**B**) Ultrastructural alterations visualized by transmission electron microscopy (TEM).

The impact of SE and CHE on cell membrane permeability was assessed by measuring β-D-galactosidase activity and protein content. Treatment with 1/4 MIC SE or CHE significantly increased extracellular β-D-galactosidase activity and protein content compared to the untreated CK group (*p* < 0.05, one-way ANOVA) (Figure 3A), demonstrating enhanced membrane permeability and loss of intracellular macromolecules. TEM analysis revealed intact cells with smooth surfaces and uniform cytoplasmic density in CK group. In contrast, SE- and CHE-treated bacteria exhibited substantial cytoplasmic reduction, decreased electron density, partial dissolution of cell wall boundaries, and cellular vacuolization (Figure 3B).

### 3.4. Transcriptome Results

#### 3.4.1. Transcriptome Data Overview

**Figure 4:**
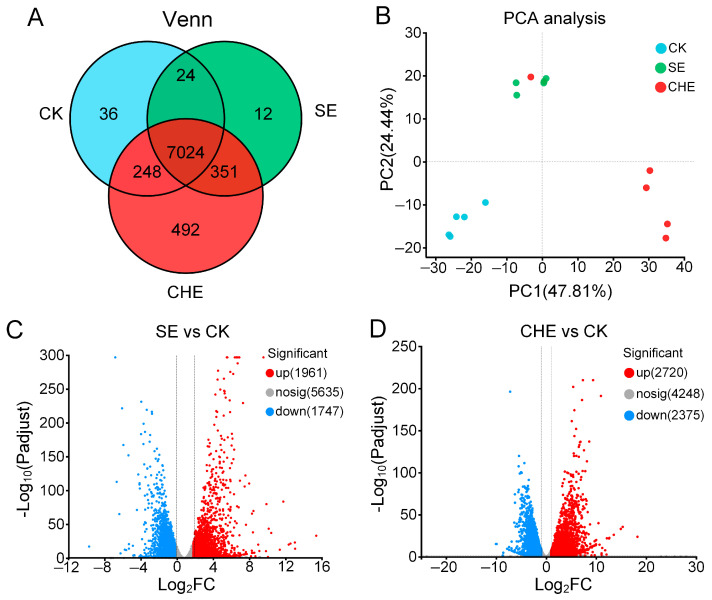
Overall differences in transcriptome of *N. seriolae* among SE, CHE and CK groups. (**A**) Venn diagram showing the unique and shared genes. CK: untreated control group. (**B**) Principal component analysis (PCA). (**C**) Volcano plots of DEGs for SE vs. CK. (**D**) Volcano plots of DEGs for CHE vs. CK. Red and blue dots represent significantly upregulated and downregulated genes, respectively, meeting the cutoff criteria of |log_2_FC| > 1 and FDR-adjusted *p* < 0.05.

Post-quality control, all samples yielded > 21.3 million clean reads with base error rates < 0.018% (acceptable threshold: <0.1%), Q20 scores > 98.4%, and genome mapping rates > 95.9% (Table 2), confirming data robustness. Venn analysis identified 7411 (SE), 8115 (CHE), and 7332 (CK) ASVs, with 363 and 740 unique ASVs in SE and CHE groups versus CK group (Figure 4A). PCA showed clear separation between treated (SE, CHE) and control (CK) groups (Figure 4B), indicating marked differences in their transcriptomic profiles. According to the DESeq2 analysis criteria (|log_2_FC| > 1, *p* < 0.05), the comparison of SE versus CK identified 1961 significantly upregulated and 1747 downregulated genes. In contrast, a greater transcriptional response was observed with CHE treatment, which resulted in 2720 upregulated and 2375 downregulated DEGs (Figure 4C,D).

#### 3.4.2. KEGG Enrichment Analysis of DEGs

**Figure 5:**
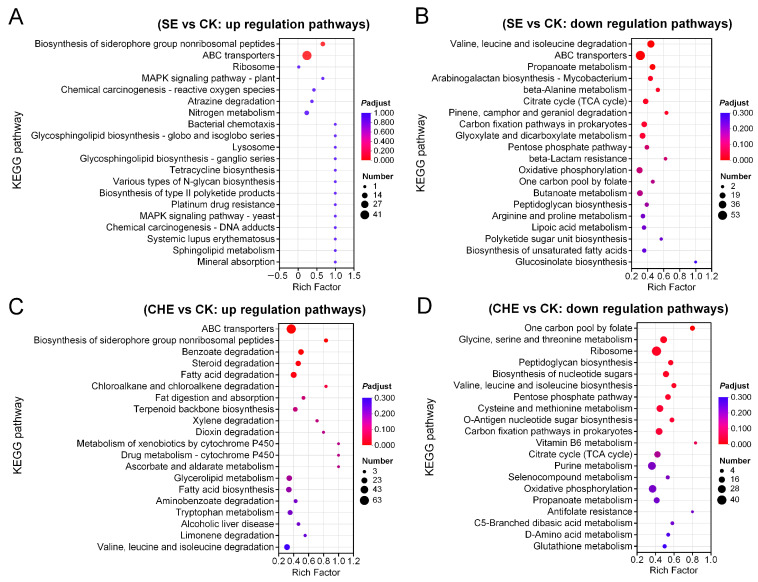
KEGG enrichment analysis of differentially expressed genes. (**A**) up regulation gene pathways for SE vs. CK; (**B**) down regulation gene pathways for SE vs. CK; (**C**) up regulation gene pathways for CHE vs. CK; (**D**) down regulation gene pathways for CHE vs. CK. CK: untreated control group.

KEGG pathway analysis of the 3708 DEGs from SE treatment and 5095 DEGs from CHE treatment identified significant dysregulation of metabolic pathways. Both treatments induced upregulation of ABC transporters and siderophore biosynthesis pathways, indicating activation of stress response and iron acquisition mechanisms. SE treatment specifically enriched glycosphingolipid and sphingolipid metabolic pathways, suggesting membrane restructuring, while CHE treatment predominantly upregulated cytochrome P450-mediated xenobiotic metabolism and fatty acid degradation pathways, reflecting enhanced detoxification capacity. Resistance-related pathways showed differential regulation: platinum drug resistance was induced by SE, whereas β-lactam resistance was downregulated by CHE (Figure 5A,C).

Conversely, both treatments consistently suppressed fundamental energy production pathways, including the citrate cycle, oxidative phosphorylation, and pentose phosphate pathway. Cell wall biosynthesis was significantly compromised through inhibition of peptidoglycan synthesis, with CHE additionally suppressing nucleotide sugar biosynthesis and SE inhibiting arabinogalactan biosynthesis. Lipid metabolism was also affected, as evidenced by downregulation of unsaturated fatty acid and glucosinolate biosynthesis pathways (Figure 5A–D).

#### 3.4.3. Clustering Analysis of Genes Enriched in Energy Metabolism Related Pathways

**Figure 6:**
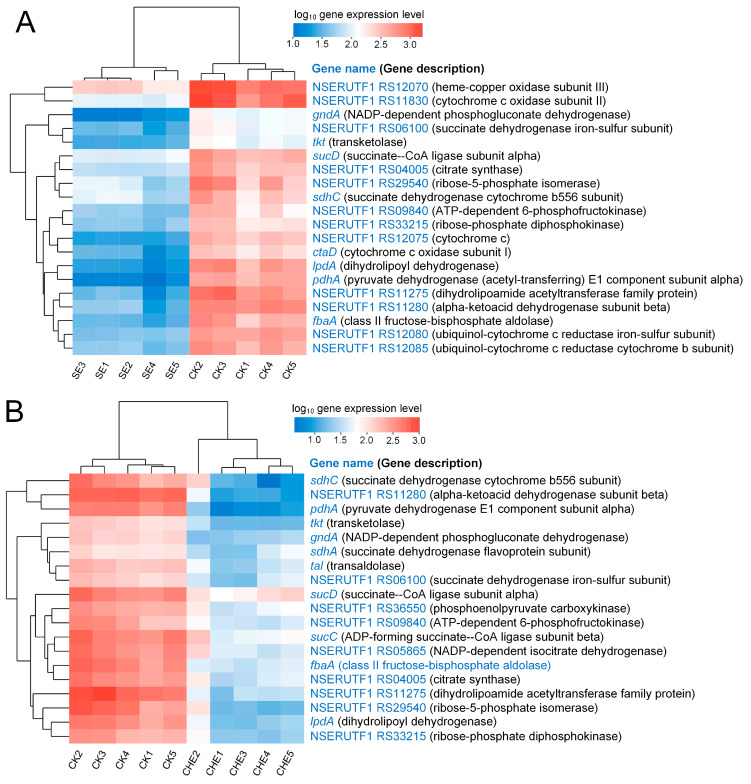
Cluster analysis of genes that annotated in energy metabolism related pathways. (**A**) down regulation genes for SE vs. CK; (**B**) down regulation genes for CHE vs. CK. CK: untreated control group.

Clustering analysis was performed to examine expression patterns of key downregulated genes involved in central carbon metabolism, including the citrate cycle, oxidative phosphorylation, and pentose phosphate pathways. The analysis revealed consistent suppression of energy metabolism genes in both SE and CHE treatment groups compared to the control. In SE-treated samples, significant downregulation was observed in genes encoding oxidative phosphorylation components, particularly cytochrome c oxidase subunits (*ctaD*, NSERUTF1_RS11830, NSERUTF1_RS12070) and electron transport chain elements (NSERUTF1_RS12080, NSERUTF1_RS12085). Citrate cycle genes including NSERUTF1_RS04005 (citrate synthase) and *sdhC* (succinate dehydrogenase) were similarly suppressed, along with pentose phosphate pathway genes *gndA* and *tkt* (Figure 6A). CHE treatment produced a more extensive suppression pattern, with additional downregulation of citrate cycle components *sucC*, *sucD*, and NSERUTF1_RS05865 (NADP-dependent isocitrate dehydrogenase). Genes encoding pyruvate dehydrogenase complex subunits (*pdhA*, *lpdA*), which link glycolysis to the citrate cycle, were downregulated in both treatments. The pentose phosphate pathway showed substantial suppression in CHE-treated cells, with reduced expression of *gndA*, *tkt*, and *tal* (Figure 6B).

#### 3.4.4. Clustering Analysis of Genes Enriched in Cell Wall Biosynthesis Related Pathways

**Figure 7:**
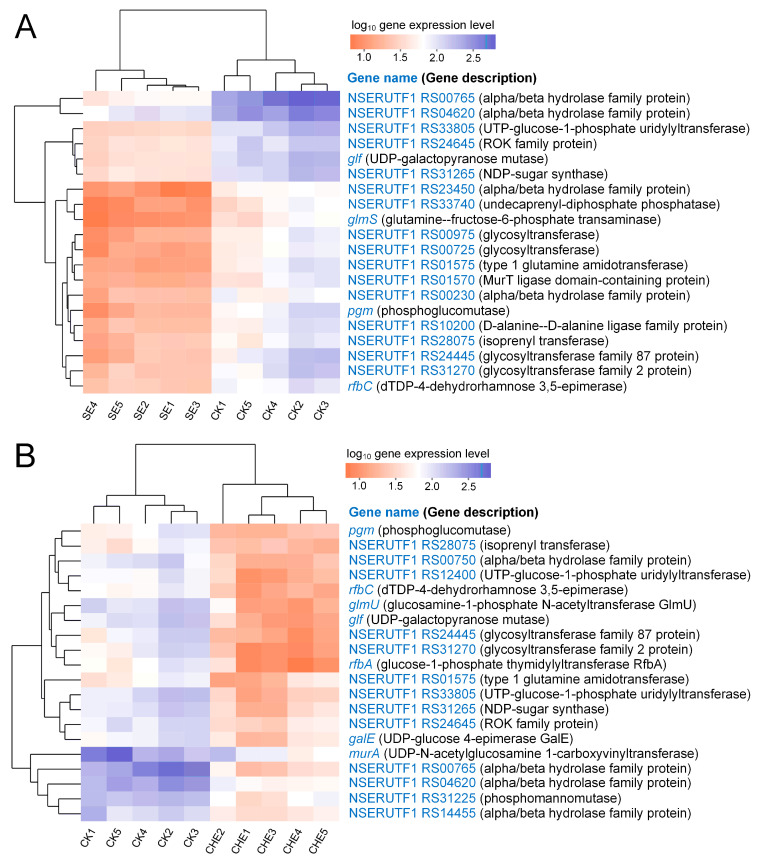
Cluster analysis of genes that annotated in cell wall biosynthesis related pathways. (**A**) down regulation genes for SE vs. CK; (**B**) down regulation genes for CHE vs. CK. CK: untreated control group.

Clustering analysis of cell wall biosynthesis-related DEGs demonstrated substantial disruption of peptidoglycan and arabinogalactan synthesis pathways (Figure 7). SE treatment impaired peptidoglycan biosynthesis through downregulation of *glmS*, involved in precursor synthesis, and NSERUTF1_RS10200 (D-alanine-D-alanine ligase), essential for peptide cross-linking. Arabinogalactan biosynthesis was affected by suppression of *glf* (UDP-galactopyranose mutase), a key enzyme for Nocardia-specific cell wall component synthesis (Figure 7A). CHE treatment induced more extensive suppression of peptidoglycan biosynthesis genes, including *murA* (UDP-N-acetylglucosamine 1-carboxyvinyltransferase) and *glmU* (glucosamine-1-phosphate N-acetyltransferase). Similar to SE treatment, CHE downregulated *glf*, indicating consistent targeting of arabinogalactan biosynthesis (Figure 7B). Both treatments suppressed multiple genes involved in nucleotide sugar precursor synthesis (NSERUTF1_RS33805, NSERUTF1_RS31265, *rfbA*, *rfbC*), further compromising cell wall integrity.

### 3.5. RT-qPCR Validation

**Figure 8:**
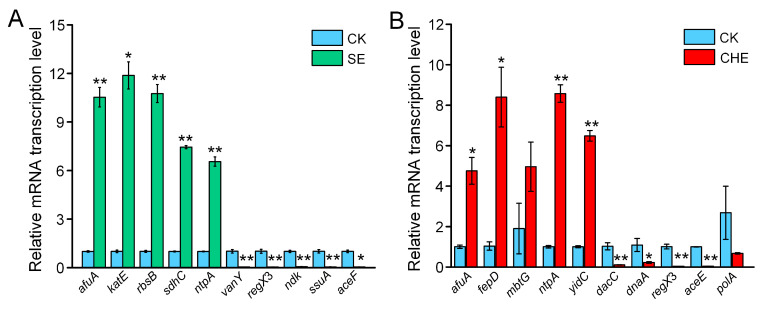
Verification of differentially expressed genes by RT-qPCR. (**A**) SE vs. CK; (**B**) CHE vs. CK. CK: untreated control group. Data present means ± standard deviations (*n =* 3). The statistical significance of the differences between the two groups are tested using an Independent *t*-test (* *p* < 0.05, ** *p* < 0.01).

Key DEGs involved in cell wall integrity, transport, virulence, and energy metabolism were validated. SE treatment significantly upregulated *afuA* (ABC transporter), *katE* (catalase), *rbsB*/*sdhC*/*ntpA* (chemotaxis) significantly increased, while downregulating *vanY* (peptidoglycan synthesis), *regX3* (two-component system), *ndk* (MAPK), and *aceF* (TCA cycle) (*p* < 0.05; Figure 8A). CHE treatment elicited similar effects: *afuA/fepD* (ABC transporters), *ntpA* (chemotaxis), and *mbtG* (siderophore biosynthesis) were upregulated, whereas *dacC* (peptidoglycan synthesis), *regX3*/*dnaA* (two-component systems), and *aceE* (TCA cycle) were downregulated (*p* < 0.05; Figure 8B). Strong correlation between RT-qPCR and RNA-seq profiles confirmed transcriptome reliability.

## 4. Discussion

### 4.1. SE and CHE Exhibit Potent Antibacterial Activity Against N. seriolae

SE and CHE, the principal benzophenanthridine alkaloids derived from *M. cordata*, have been extensively investigated for their antimicrobial properties [9,10,11]. This study confirms their substantial in vitro antibacterial efficacy against *N. seriolae*, with CHE and SE demonstrating enhanced potency as reflected by its lower MICs and MBCs. These values are comparable to those of conventional antibiotics such as flumequine, amoxicillin and florfenicol [25], underscoring their potential as therapeutic alternatives. Growth kinetic analysis revealed a distinct concentration-dependent inhibition pattern, further validating their antibacterial activity. These findings are consistent with the established broad-spectrum antibacterial properties of benzophenanthridine alkaloids [26]. SE has been documented to effectively inhibit various Gram-positive and Gram-negative pathogens, including *S. aureus* and *A. dhakensis* [27,28]. Similarly, CHE has demonstrated strong activity against multiple bacterial species, with its mechanism potentially involving membrane disruption and metabolic interference [10,29]. The antibacterial potency of both compounds against *N. seriolae* appears comparable to conventional antibiotics used in aquaculture, suggesting their potential as viable alternatives in disease management.

The in vivo efficacy of SE and CHE was clearly demonstrated through their ability to control *N. seriolae* infection in largemouth bass. Dietary supplementation with either compound significantly reduced the bacterial load in hepatic tissues and markedly enhanced host survival rates. This finding consistent with previous studies in Channel Catfish (*Ictalurus punctatus*) and Pacific white shrimp (*Litopenaeus vannamei*) [8,30]. Notably, both treatments effectively suppressed the formation of characteristic granulomatous lesions across multiple organs, demonstrating considerable mitigation of nocardiosis-associated pathology. However, some mortality persisted despite this clear suppression of pathology. This indicates that the alkaloids controlled established infections and halted tissue damage progression. Yet, they may have been less effective in reversing early systemic dissemination or severe organ damage occurring before treatment. This protective efficacy was mediated primarily through direct antibacterial action. It positions these compounds as promising targeted therapeutics for aquaculture. The dual capacity to inhibit bacterial proliferation while minimizing tissue damage represents a crucial criterion for effective aquaculture chemotherapeutics. Previous research on *M. cordata* extracts has similarly shown effective control of bacterial infections in various fish species without compromising growth performance [31,32]. Together, our findings underscore the therapeutic value of SE and CHE, while also suggesting that their greatest benefit may come from prophylactic use or early intervention. It is, however, imperative to balance efficacy with safety considerations. A study has demonstrated that the *M. cordata* extract exhibits a clear dose-dependent toxicological profile in rodents [33], underscoring the critical importance of confining the use of these compounds to a defined safe therapeutic window. Therefore, future studies are warranted to delineate the compound-specific chronic toxicity and residue kinetics in aquatic species, which is essential to fully establish their safety for aquaculture applications.

### 4.2. SE and CHE Disrupt Cellular Structure and Suppress Cell Wall Biosynthesis in N. seriolae

The cell wall and membrane serve as the primary barrier against antimicrobial agents, playing crucial roles in maintaining bacterial structural integrity and regulating nutrient transport [34]. In this study, we demonstrate that SE and CHE induce comprehensive damage to the bacterial cell envelope of *N. seriolae.* This aligns with their previously reported mechanisms against Gram-positive pathogens like *S. aureus* and *S. agalactiae* [9,10]. TEM analysis revealed substantial cytoplasmic reduction and dissolution of cell wall boundaries, while biochemical assays indicated markedly increased extracellular β-D-galactosidase activity and protein content. These results together confirm severe impairment of membrane and wall integrity. These morphological alterations were supported by the transcriptomic data. The data show significant downregulation of key genes involved in peptidoglycan biosynthesis, including *glmS* (precursor synthesis) and *NSERUTF1_RS10200* (D-alanine-D-alanine ligase). Notably, both compounds suppressed *glf* (UDP-galactopyranose mutase), an essential enzyme for arabinogalactan biosynthesis—a characteristic component of the cell wall of *Nocardia* and *Mycobacteria* [35]. Furthermore, CHE specifically inhibited *murA*, which encodes the first committed enzyme in peptidoglycan synthesis [36], whereas SE uniquely affected genes involved in peptidoglycan amidation. Taken together, these findings suggest SE and CHE may act via a multi-target strategy, which involves disrupting both universal peptidoglycan synthesis and pathogen-specific cell wall components.

The cell wall-disrupting effects observed in this study are consistent with previously documented mechanisms of benzophenanthridine alkaloids. Specifically, SE has been demonstrated to compromise the cell envelope integrity of *S. aureus* by increasing membrane permeability [9]. In parallel, CHE disrupts the cell wall of *Xanthomonas oryzae* pv. *Oryzae* by inhibiting the synthesis of peptidoglycan as well as pyrimidine and purine nucleotides [29]. Further supporting these findings, SE has been reported to impair the cell membrane integrity of *Providencia rettgeri*, as indicated by reduced intracellular ATP levels and cytoplasmic acidification [37]. Similarly, CHE increases membrane permeability in *S. agalactiae*, leading to the leakage of intracellular components such as ATP, alkaline phosphatase, and key electrolytes including Na^+^, K^+^, Ca^2+^, and Mg^2+^ [10]. The consistency between these literature findings and our experimental data strongly supports the conclusion that disrupting the cell wall and membrane is a key mechanism of SE and CHE against *N. seriolae.*

### 4.3. SE and CHE Impair Energy Metabolism in N. seriolae

Transcriptomic analysis revealed that the antibacterial effect was associated with a broad transcriptional suppression of energy metabolism pathways in *N. seriolae*. Hierarchical clustering demonstrated consistent downregulation of genes encoding essential enzymes in central carbon metabolic pathways, including the citrate cycle (e.g., citrate synthase and succinate dehydrogenase), oxidative phosphorylation (e.g., cytochrome c oxidase subunits and ubiquinol-cytochrome c reductase), and the pentose phosphate pathway (e.g., phosphogluconate dehydrogenase and transketolase). The coordinated suppression of these core energy-producing pathways is predicted to cause severe ATP depletion and metabolic dysfunction, ultimately leading to bacterial growth arrest [38]. The observed inhibition of energy metabolism aligns with previously reported effects of benzophenanthridine alkaloids. For instance, a previous study revealed that SE primarily disrupts amino acid synthesis and energy metabolism pathway such as phenylalanine metabolism, glycolysis and citrate cycle in *Enterobacter* *cloacae* [39]. Similarly, in *Xanthomonas oryzae*, CHE was found to inhibit carbohydrate metabolism and sugar phosphorylation, while disrupting nucleotide synthesis and cell division, ultimately impairing energy production and bacterial viability [29]. Furthermore, both SE and CHE significantly downregulated pyruvate dehydrogenase complex genes (*pdhA*, *lpdA*). This effect likely blocks the metabolic link between glycolysis and the citrate cycle and creating a catalytic bottleneck. Bacterial energy metabolism is closely linked not only to growth but also to adaptive stress responses and virulence expression [40]. Therefore, impairing ATP synthesis and membrane energetics may compromise essential physiological processes such as efflux pump activity, cell division, and envelope integrity maintenance [41]. Such impairment could thereby enhance the lethality of SE and CHE. Collectively, the multi-pathway suppression of energy metabolism, combined with the demonstrated cell envelope damage, underscores a multi-target antibacterial mode of action. This provides a comprehensive mechanistic insight into the efficacy of SE and CHE against *N. seriolae*.

## 5. Conclusions

This study confirms that *M. cordata* alkaloids SE and CHE possess significant efficacy against *N. seriolae* through integrated in vitro and in vivo assays. They exert a multi-target antibacterial mechanism, synergistically disrupting cell envelope integrity and inhibiting central energy metabolism pathways, ultimately resulting in bacterial death. These findings support the development of these alkaloids as eco-friendly alternatives for controlling nocardiosis in aquaculture.

## Figures and Tables

**Table 1:** Real-time PCR primer sequences.

Primer Name	Sequence (5′–3′)	Tm Value
*afuA*	F: GGCCAAGACCAAGGAGAACA	59.89
	R: TCAGGAACTTCTGGGCGTTG	60.25
*urtA*	F: AGCAATACCAGCTACGAGGC	59.89
	R: TTGATTGCCCTCGAAACCGA	59.96
*katE*	F: TGGTTACCGGTGATGTGAGC	60.04
	R: GGTGTAGAACTTCACCGCGA	60.04
*sdhC*	F: CATCGCGCTGTATCTGGTCT	59.97
	R: GCCAGGACATAGAACACCGT	59.75
*ntpA*	F: ACGCGGATGTGATCGTCTAC	59.97
	R: GCCATATTGGAGGTGTTGGC	59.25
*tauA*	F: GGCGAATACGCCTACCTGAA	59.90
	R: TGGCAAGACCCTGAATCTCG	59.75
*mbtG*	F: CTGGACCTTTTCGACACCGA	59.97
	R: CCAGATTCGGCAGGTAGAGC	60.25
*RegX3*	F: CTGCTCGATCTCATGCTCCC	60.32
	R: CACCTTGTCGATCTCGCTGT	60.11
*acd*	F: TGCTGGGTCTGACAAAGACG	60.25
	R: TGGTAGGGGATCGACAGCAT	60.40
*ndk*	F: AGATCATCACCCGCATCGAG	59.68
	R: ATTCGATGAGCGAGCCGAAG	60.60
*pstS*	F: GTGAATCTCAAGCGCAGCAG	59.90
	R: GGTCCATGGCGTTCTTCTGA	60.04
16S	F: AGAGTTTGATCCTGGCTCAG	55.40
	R: TACGGCTACCTTGTTACGACTT	55.81

**Table 2:** Statistics of transcriptome sequencing data.

Sample Name	Raw Error Rate (%)	Clean Reads	Clean Q20 (%)	Genome Mapped Ratio (%)
SE1	0.014	24, 397, 522	98.01	96.18
SE2	0.014	25, 413, 870	98.02	96.44
SE3	0.014	24, 691, 366	97.95	95.93
SE4	0.013	23, 601, 956	98.37	98.08
SE5	0.013	24, 801, 536	98.38	97.39
CHE1	0.017	27, 114, 160	96.75	98.11
CHE2	0.014	21, 313, 624	98.21	96.48
CHE3	0.018	34, 308, 280	96.30	96.95
CHE4	0.016	23, 894, 644	97.37	97.36
CHE5	0.016	28, 653, 500	97.62	98.37
CK1	0.013	25, 211, 102	98.35	98.27
CK2	0.014	24, 457, 136	98.30	96.48
CK3	0.014	25, 376, 278	98.25	96.83
CK4	0.014	28, 698, 816	98.28	97.20
CK5	0.013	23, 629, 998	98.42	97.55

## Data Availability

The raw RNA-seq sequencing data generated in this study have been deposited in the NCBI Sequence Read Archive (SRA) under the BioProject accession number PRJNA1372400.
